# Effects of Consuming Xylitol on Gut Microbiota and Lipid Metabolism in Mice

**DOI:** 10.3390/nu9070756

**Published:** 2017-07-14

**Authors:** Takashi Uebanso, Saki Kano, Ayumi Yoshimoto, Chisato Naito, Takaaki Shimohata, Kazuaki Mawatari, Akira Takahashi

**Affiliations:** Department of Preventive Environment and Nutrition, Institute of Biomedical Sciences, Tokushima University Graduate School, 3-18-15, Tokushima 770-8503, Japan; c201302046@tokushima-u.ac.jp (S.K.); sea.by15.koko@gmail.com (A.Y.); chii.cham.0701@gmail.com (C.N.); shimohata@tokushima-u.ac.jp (T.S.); mawatari@tokushima-u.ac.jp (K.M.); akiratak@tokushima-u.ac.jp (A.T.)

**Keywords:** xylitol, triglyceride, cholesterol, *Streptococcus mutans*, denaturing gradient gel electrophoresis (DGGE), capillary electrophoresis–mass spectrometry (CE–MS), caries

## Abstract

The sugar alcohol xylitol inhibits the growth of some bacterial species including *Streptococcus mutans*. It is used as a food additive to prevent caries. We previously showed that 1.5–4.0 g/kg body weight/day xylitol as part of a high-fat diet (HFD) improved lipid metabolism in rats. However, the effects of lower daily doses of dietary xylitol on gut microbiota and lipid metabolism are unclear. We examined the effect of 40 and 200 mg/kg body weight/day xylitol intake on gut microbiota and lipid metabolism in mice. Bacterial compositions were characterized by denaturing gradient gel electrophoresis and targeted real-time PCR. Luminal metabolites were determined by capillary electrophoresis electrospray ionization time-of-flight mass spectrometry. Plasma lipid parameters and glucose tolerance were examined. Dietary supplementation with low- or medium-dose xylitol (40 or 194 mg/kg body weight/day, respectively) significantly altered the fecal microbiota composition in mice. Relative to mice not fed xylitol, the addition of medium-dose xylitol to a regular and HFD in experimental mice reduced the abundance of fecal *Bacteroidetes phylum* and the genus *Barnesiella*, whereas the abundance of *Firmicutes phylum* and the genus *Prevotella* was increased in mice fed an HFD with medium-dose dietary xylitol. Body composition, hepatic and serum lipid parameters, oral glucose tolerance, and luminal metabolites were unaffected by xylitol consumption. In mice, 40 and 194 mg/kg body weight/day xylitol in the diet induced gradual changes in gut microbiota but not in lipid metabolism.

## 1. Introduction

Gut microbiota form many bioactive metabolites from dietary components which can regulate host metabolism [1,2,3,4,5]. For example, an improvement in glucose metabolism induced by dietary fiber is associated with the increased abundance of *Prevotella* [2]. Similarly, some food derivatives and food additives can affect host metabolism after interactions with gut microbiota [1,5].

Xylitol has a caries preventative effect via its capacity to inhibit the growth of *Streptococcus mutans* [6]. Dietary xylitol, metabolized into D-xylulose-5-phosphate, activates the carbohydrate response element binding protein (ChREBP) [7]. We previously reported that dietary xylitol combined with a high-fat diet (HFD) induced hepatic lipogenic gene expression via ChREBP mRNA expression [8]. In this report, we revealed that xylitol can improve HFD-induced hypertriglyceridemia and hypercholesterolemia with cecum enlargement in mice. In another report, the administration of a 2.5–10% xylitol solution reduced serum cholesterol and low density lipoprotein-cholesterol in diabetic mice [9]. Moreover, mice supplemented with 5% xylitol and 0.05% daidzein in their diet had a lower serum cholesterol versus mice fed a diet containing daidzein alone [10]; xylitol also contributed to the relative reduction of the genera *Bacteroides* and *Clostridium* in gut microbiota. *Clostridium* genus clusters *XI* and *XIVa* participate in the conversion of primary bile acids to secondary bile acids [11]. Cholic acid, one of the primary bile acids, promote cholesterol absorption [12]. Moreover, alteration of the bile acids composition regulates lipid and energy metabolism through the activation of the farnesoid *X* receptor (FXR) or G-protein-coupled receptors (GPCRs), such as TGR5 [13,14]. On the other hand, the gut microbiota suppress fat accumulation via the short-chain fatty acid production from dietary fiber [15]. Taken together, dietary xylitol is able to improve hyperlipidemia and modify gut microbiota. However, at least 1.5 g/kg body weight/day of dietary xylitol was given in those studies [8,9,10]. The effects of daily dietary xylitol at relatively lower doses on gut microbiota and lipid metabolism are unclear. Over the past decade, pure xylitol and xylitol comestible products (e.g., gums and candies) have been commercially available to the general public. In addition, some infants are given xylitol tablets for the health of their teeth. Infants can potentially ingest more xylitol, up to 200 mg/kg body weight/day (commercially recommended xylitol tablets), than that of adults. In other reports, 150–300 mg xylitol/kg body weight/day have been used for preventing caries in schoolchildren [16,17]. Because development and expansion of the gut bacterial community occurs relatively slowly during early childhood [18], environmental factors could more strongly affect gut microbes in children than in adults. In the present study, our goal was to estimate the effect of feeding low-dose xylitol on gut microbiota and lipid metabolism in mice from an early stage of life.

## 2. Materials and Methods

### 2.1. Animals

Seven-day pregnant female C57Bl/6J mice were purchased from a local breeding colony (Charles River Japan, Yokohama, Japan) and their offspring—male pups only—were used in experiments 1 and 2 of this study. Mice were housed in cages maintained at constant temperature (23 ± 2 °C) and relative humidity (65–75%) with a 12-h light/dark cycle (8:00–20:00). In experiment 1, all three-week-old males were fed the control diet (CD, AIN93G, Oriental Yeast, Osaka, Japan) formulated for rapid growth for 16 weeks, during which time they were divided into three groups as follows: control diet (CD) group, with free access to distilled water (CD, *n* = 5); low-dose xylitol group, were given xylitol solution of 40 mg/kg body weight/day (CD-LX, *n* = 5); and a medium-dose xylitol group, were given xylitol solution of 200 mg/kg body weight/day (CD-MX, *n* = 5). In experiment 2, three-week-old male mice were fed a high-fat diet (HFD32, Japan Crea, Osaka, Japan) for 18 weeks, during which time they were divided into two groups as follows: high-fat diet (HFD) group with free access to distilled water (HFD, *n* = 5) and an HFD with a medium-dose xylitol group, were given xylitol solution of 200 mg/kg body weight/day (HFD-MX, *n* = 6). Body weight and fluid intake were measured three or four times weekly. We used to control the xylitol consumption of mice using pair-feeding like method. The xylitol concentration was calculated on the basis of daily fluid intake and body weight; adjustments to the concentration of xylitol in the drinking water were made every 1–2 days to regulate xylitol consumption. In the fecal microbiota transplantation (FMT) experiment, six-week-old male mice were treated with a cocktail of broad spectrum antibiotics (1 g/L ampicillin, neomycin, and metronidazole and 0.5 g/L vancomycin) in their drinking water for three weeks [19]. FMT was performed to transplant gut microbiota from donor mice fed an HFD, with or without xylitol (HFD-MX-FMT and HFD-FMT, respectively) to antibiotic-treated recipient mice as has been reported previously with slight modifications [19]. The transplantation procedure was performed every 3 days, twice per experiment. After an FMT, mice were maintained on HFD for eight weeks. All mice were euthanized; blood was collected in addition to ceca, cecal contents, feces, and liver tissue. The University of Tokushima Animal Use Committee approved the study (T14010), and mice were maintained according to the National Institutes of Health Guide for the Care and Use of Laboratory Animals.

### 2.2. Oral Glucose Tolerance Test

At week 16, mice fed the HFD and HFD-MX were fasted for 16 h and subsequently administered 2 g glucose/kg body weight orally to test their glucose tolerance. Blood samples taken from the tail vein at indicated times were used to determine plasma glucose concentration (Glucose Pilot, IWAI CHEMICALS COMPANY, Tokyo, Japan).

### 2.3. Extraction of Genomic DNA and Quantitative PCR

Genomic DNA from fecal and cecal content samples were isolated using the FavorPrep Stool DNA Isolation Mini Kit (FAVORGEN Biotech Corp., Ping-Tung, Taiwan) in accordance with the manufacturer’s protocol. The relative abundance of each target’s bacterial 16S rRNA gene sequence (see primer sequences in Table 1) was calculated by normalization to the amount of amplified product from all bacteria 16S rRNA gene copy numbers. 

### 2.4. PCR-DGGE Analysis

Denaturing gradient gel electrophoresis (DGGE) was performed as previously described [24] using the DCode^TM^ Universal Mutation Detection System instrument and model 475 gradient former according to the manufacturer’s instructions (Bio-Rad Labs, Hercules, CA, USA). The V2–V3 region of the 16S rRNA genes (positions 339–539 in the *Escherichia coli* gene) of bacteria in gut samples was amplified with the primers HDA1-GC and HDA2. PCR reaction mixtures and the amplification program were the same as described previously [24]. The denaturing gradient was formed with two 8% acrylamide (acrylamide-bis 37.5:1) with denaturing gradients ranging from 20–80% for analysis of the amplified 16S rRNA fragments. The 100% denaturant solution contained 40% (*v*/*v*) deionized formamide and 7 M urea. PCR product (40 μL) was mixed with 40 μL dye before loading. Gels were run in 0.5× Tris/Acetate/EDTA buffer at 60 °C for 5.2 h at 180 V, 210 mA, stained with Gel Star (Lonza Japan, Tokyo, Japan) for 30 min, and analyzed by ChemiDoc MP (Bio-Rad, Hercules, CA, USA). Image Lab software, version 5.0 (Bio-Rad) was used for the identification of bands and normalization of band patterns from DGGE gels.

### 2.5. Determination of Bacterial Strain by Sequence Analysis 

Specific bands from DGGE gels were excised for DNA extraction, mashed, and incubated overnight in a diffusion buffer (0.5 M ammonium acetate, 1 mM EDTA, 0.1% SDS, 15 mM magnesium acetate). DNA was purified by the standard ethanol precipitation method. The V2–V3 region of the 16S rRNA genes were amplified by PCR, and purified DNA was used as a template. PCR products were cloned into the pCR2.1-TOPO vector (Invitrogen, Carlsbad, CA, USA), sequenced, and the bacterial genus was identified by BLAST.

### 2.6. Plasma and Hepatic Lipid Concentrations

Hepatic lipids were extracted and measured as previously described [25]. Plasma and liver triglycerides (TG) and total cholesterol concentration were measured by using Triglyceride-E and Cholesterol-E tests (Wako Pure Chemical Industries, Osaka, Japan), respectively. 

### 2.7. RNA Preparation and Quantitative Reverse Transcriptase PCR

Extraction of total RNA, cDNA synthesis, and real-time PCR analysis were performed as described previously [25]. The relative abundance of each target transcript was calculated by normalization to the amount of amplified product from constitutively expressed β-actin mRNA (see primer sequences in Table 1).

### 2.8. Metabolome Analysis of Cecum Luminal Content by Capillary Electrophoresis Electrospray Ionization Time-of-Flight Mass Spectrometry

The cecum luminal content was immediately frozen in liquid nitrogen and stored at −80 °C until metabolite extraction. Sample tissues were weighed and completely homogenized in 0.5 mL ice-cold methanol containing 50 μM methionine sulfone and camphor-10-sulfonic acid as internal standards. The homogenates were mixed with 0.5 mL chloroform and 0.2 mL ice-cold Milli-Q water. After centrifugation at 2300× *g* for 5 min, the supernatant was centrifugally filtrated through 5-kDa cut-off filters (Millipore, Bedford, MA, USA) at 9100× *g* for 4–5 h to remove proteins. The filtrate was centrifugally concentrated in a vacuum evaporator, dissolved with Milli-Q water, and analyzed by capillary electrophoresis electrospray ionization time-of-flight mass spectrometry (CE-TOFMS).

CE-TOFMS analysis was performed using an Agilent CE system combined with a TOFMS (Agilent Technologies, Palo Alto, CA, USA) as reported by previously [24,26,27]. Each metabolite was identified based on a reference which containing internal standards including 110 metabolites (H3304-1002, Human Metabolome Technology (HMT), Inc., Tsuruoka, Japan) to *m*/*z* and migration time, and quantified by peak area.

### 2.9. Statistical Analyses

Data are expressed as means ± standard errors of the mean (SEM). A significant difference between groups was assessed via an unpaired two-tailed *t*-test in experiment 2 and FMT experiment. For comparisons among more than three groups, we employed analysis of variance (ANOVA) or the Kruskal-Wallis test in experiment 1. When a significant difference was found by ANOVA or the Kruskal-Wallis test, post hoc analyses were performed using the Tukey-Kramer protected least significant difference test. Concentration-dependent effects were identified via linear regression analysis. Spearman’s rank correlation coefficient was used to calculate correlation coefficients between selected variables. Differences were considered significant at *p* < 0.05. Statistical analyses were performed using Mass Profiler Professional and Excel-Toukei 2006 (SSRI, Tokyo, Japan).

## 3. Results

**Figure 1:**
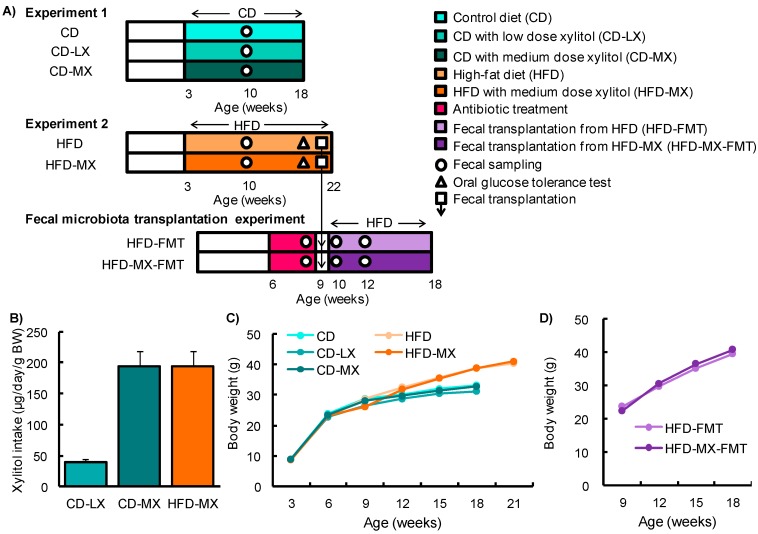
Experimental design and changes in body weight in mice fed xylitol. Study design for experiment 1 and 2 and the fecal transplantation experiment (**A**). Xylitol consumption during experiments (**B**). Changes in body weight (BW) throughout experiment 1 and 2 (**C**) and during the fecal transplantation experiment (**D**). Data represent the mean ± SEM (*n* = 5–6).

**Figure 2:**
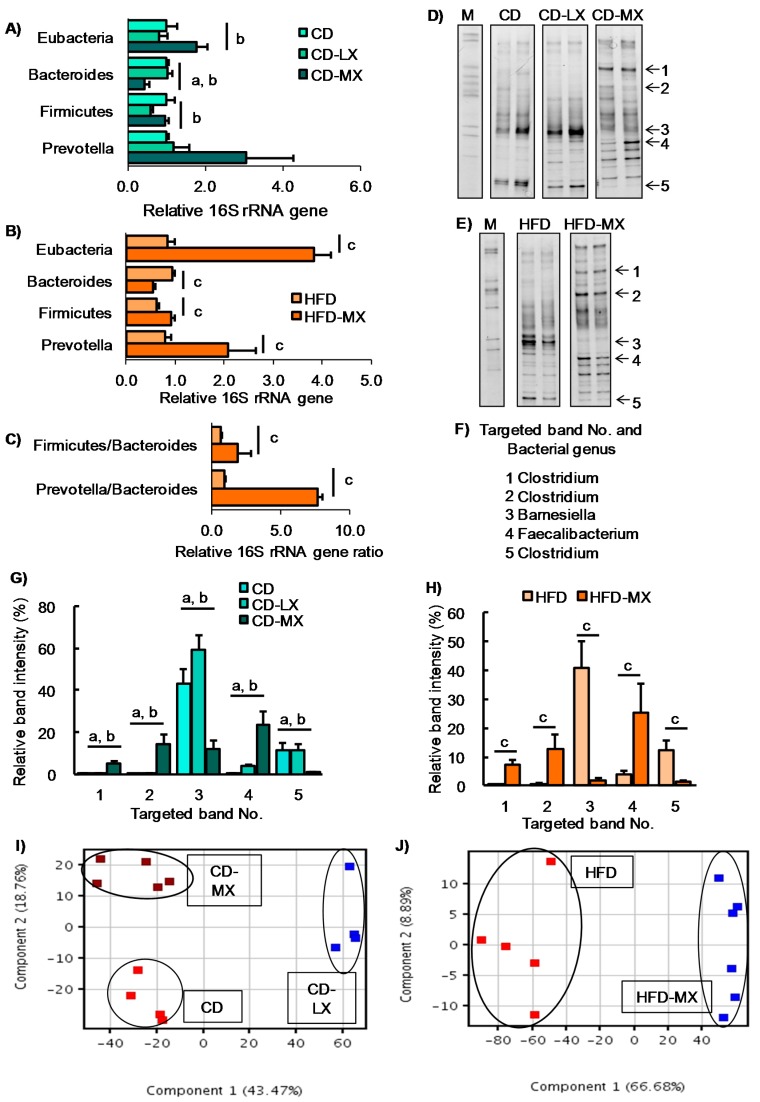
Changes in the fecal microbiota of mice fed xylitol. An abundance of specific bacterial phylum or genus and ratio after seven weeks of xylitol supplementation using specific primer set (Table 1) (**A**–**C**). Band image of DGGE analysis of DNA from feces after seven weeks of xylitol exposure with CD (**D**) or HFD (**E**). Identified five bacterial genus (No. 1–5) from DGGE band (**F**). Relative band density of identified five bacterial genus from feces after seven weeks of xylitol exposure with CD (**G**) or HFD (**H**). Two-dimensional principal component analysis plot of DGGE band pattern in mice fed xylitol with CD (**I**) or HFD (**J**). Data represent the mean ± SEM (*n* = 5–6). a: *p* < 0.05 between CD and CD-MX. b: *p* < 0.05 between CD-LX and CD-MX. c: *p* < 0.05 between HFD and HFD-MX.

To elucidate the effect of consuming low-dose xylitol on gut microbiota and lipid metabolism, the mean xylitol dosage administered to mice after weaning was 40 *±* 5 mg/kg body weight/day (CD-LX), 194 ± 24 mg/kg body weight/day (CD-MX), and 194 ± 25 mg/kg body weight/day (HFD-MX) (Figure 1A,B). During the treatment periods, body weight, relative epididymal fat weight per body weight, relative liver weight per body weight, and relative cecum weight per body weight were not different between the xylitol-fed groups and the control group of mice in experiment 1 and 2 (Figure 1C,D, and Table 2). The relative amount of total fecal bacteria to fecal DNA displayed a trend towards an increase in the feces of CD-MX mice and was significantly increased in the feces of HFD-MX mice when compared with control mice (Figure 2A,B). In contrast, *Bacteroides*, a phylum of bacteria, was reduced in both MX mice fed a CD or HFD (Figure 2A,B). In addition, the combination of an HFD and ingestion of a medium-dose xylitol solution showed that an increased amount of *Firmicutes phylum*, the *Prevotella genus*, and the relative ratio of *Firmicutes*/*Bacteroides* and *Prevotella*/*Bacteroides* than those of HFD fed control mice (Figure 2B,C). To explore in detail the microbiome bacterial composition, we carried out DGGE analysis. We identified five genera, which included two species of *Clostridium* and a *Faecalibaculum* genus which were increased in the MX mice and one from both the *Clostridium* and *Barnesiella genera* which were reduced in the MX mice; different analysis bands were significantly different (Figure 2D–H). Principal component analysis (PCA) allowed us to clearly distinguish among groups based on dietary xylitol exposure, regardless of the control or HFD (Figure 2I,J). Continuous daily consumption of 40 or 194 mg xylitol after weaning induced different populations of gut microbiota in the feces of mice.

**Figure 3:**
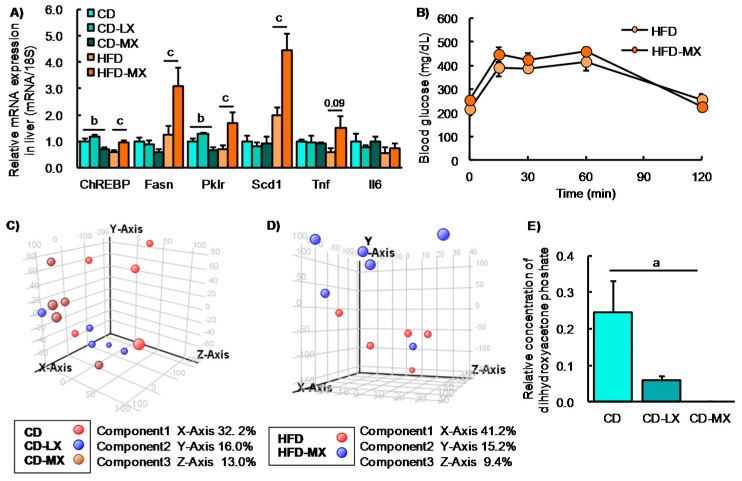
Hepatic gene expression, oral glucose tolerance test, and luminal metabolite in xylitol-fed mice. Relative hepatic gene expression involved in lipid metabolism in mice fed xylitol (**A**). Changes in blood glucose levels during an oral glucose tolerance test (OGTT) in mice fed xylitol with the HFD (**B**). Principle component analysis of 94 luminal metabolites in mice supplemented with xylitol and the CD (**C**) or the HFD (**D**). Changes in relative concentration of luminal dihydroxyacetone phosphate in mice supplemented with xylitol and the CD (**E**). Fasn: fatty acid synthase, Pklr: pyruvate kinase liver and red blood cell. Data represent the mean ± SEM (*n* = 5–6). a: *p* < 0.05 between CD and CD-MX. b: *p* < 0.05 between CD-LX and CD-MX. c: *p* < 0.05 between HFD and HFD-MX.

Our study and others report that a high dose of xylitol improved hyperlipidemia in mice fed an HFD and in diabetic mice [8,9,10]. To reveal the effect of a low dose of xylitol on lipid metabolism, we investigated cholesterol and triglyceride concentrations in the liver and serum, parameters which were not different among the three groups of mice maintained on the control diet (Table 2). In contrast, an HFD induced hypertriglyceridemia and hypercholesterolemia in the liver, but xylitol supplementation did not ameliorate dyslipidemia (Table 2). We also found that hepatic ChREBP and the expression of its target genes were increased in HFD-MX mice compared with control mice (Figure 3A) as was reported in a previous study [8]. In addition, we investigated glucose tolerance in mice fed an HFD because two reports have shown an abundance of several different species of the genus *Prevotera* that are linked with glucose intolerance or insulin resistance in humans and mice [2,28]. We could not detect any changes in glucose tolerance, as well as the expression of inflammation-related genes, in mice fed the HFD with or without dietary xylitol supplementation (Figure 3A,B).

To further investigate the effects of xylitol intake on luminal metabolites, we conducted a CE-MS analysis. We identified 94 metabolites from a metabolite list provided by HMT. From the PCA plot, we were unable to distinguish any metabolite patterns among the groups of mice in experiment 1 fed the AIN93G diet with or without supplemental dietary xylitol in their drinking water (Figure 3C,D). Only dihydroxyacetone phosphate concentration was different between CD and CD-MX groups. These results suggest that the changes in luminal content microbiota in xylitol supplemented groups had little, if any, effect on overall metabolism.

**Figure 4:**
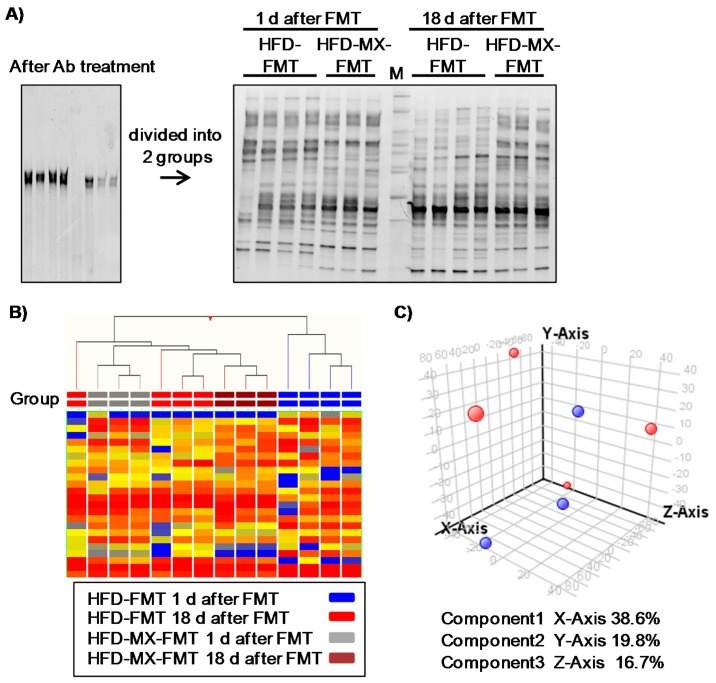
Changes in fecal microbiota and luminal metabolites of recipient mice in fecal transplantation experiment. Fecal transplantations were performed from donor mice fed the HFD with or without xylitol to antibiotic (Ab)-treated recipient mice (HFD-FMT and HFD-MX-FMT). Band patterns (**A**) and hierarchical clustering of band patterns (**B**) in feces 16S V2–V3 rRNA composition in HFD-FMT and HFD-MX-FMT mice. Ab treatment reduced fecal bacteria (left band pattern) and gradual changes in the composition of fecal bacteria in the HFD-FMT and HFD-MX-FMT mice from 1 day after FT to 18 day after FMT. M: Marker. Principle component analysis of luminal metabolites in HFD-FMT (red) and HFD-MX-FMT mice (blue). (**C**). *n* = 3–4.

Finally, we attempted to detect microbiota-dependent effects of xylitol feeding in mice fed an HFD via FMT. One day after the final transplantation, the microbiota was clearly different between the mice that were recipients of feces transplanted from mice fed an HFD (HFD-FMT) and fed an HFD with medium-dose xylitol (HFD-MX-FMT) (Figure 4A,B). These perceptible differences between the two groups disappeared 18 day after the transplantation (Figure 4A,B). No changes in luminal metabolites, body weight, and relative tissue weight between HFD-FMT and HFD-MX-FMT mice were detected (Figure 4C, Table 3). These results indicate that changes in the fecal microbiota of mice fed xylitol are transient and likely continuous xylitol supplementation is necessary to sustain the changes observed. Interestingly, serum cholesterol in the HFD-MX-FMT mice was slightly, but significantly, higher than that of the HFD-FMT mice (Table 3). This suggests that changes in the composition of microbiota induced by dietary xylitol increase serum cholesterol. 

## 4. Discussion and Conclusions

In this study, we showed that the administration of xylitol at 40 and 194 mg/kg body weight/day significantly altered gut microbiota in mice. In particular, we noted the relative abundance of the *Bacteroidetes phylum* was reduced in mice in the CD-MX and HFD-MX groups, indicating that xylitol suppressed the growth of some bacterium, including the genus *Barnesiella* in mice fed either CD or HFD. In contrast, the relative abundance of *Firmicutes phylum* and the genus *Prevotella* were increased in the HFD-MX group. Contrary to the significant alteration of microbiota, body composition, lipid parameters, and luminal metabolites were not different between groups, regardless of xylitol consumption. 

The improvement of glucose tolerance observed with increased dietary fiber intake is linked with a higher abundance of the genus *Prevotella* [2]. In contrast, the abundance of *Prevotella copri* was positively associated with microbial branched-chain amino acid (BCAA) biosynthesis in the gut and insulin resistance with a soy protein diet which contained a low level of BCAAs [28]. Our present study showed an increased abundance of *Prevotella* and an increase in the *Prevotella*/*Bacteroidetes* ratio, but no differences were observed in glucose tolerance or luminal BCAA concentrations between the HFD and HFD-MX groups. Because the mice were fed a diet containing casein, which has as the protein source a high BCAA content, we were unable to detect any changes in the luminal BCAA concentrations. These results suggest that changes in the bacterial composition and the supply of dietary components modulates host metabolism in a coordinated manner. 

An amount of dietary indigestible fiber and gut microbiota which digest fiber regulates cecum weight [29,30]. In the experiment 1, we used AIN93G as a control diet which contains more fiber (5%) than the HFD which used in experiment 2 (2.9%). In the present study, xylitol feeding did not affect cecum weight, therefore a difference in the amount of dietary fiber might affect cecum weight. 

In our study, daily supplemental dietary xylitol of 194 but not 40 mg/kg body weight induced significant changes in microbiota for the genera *Barnesiella,* which was reduced, and *Feacalibaculum,* which was increased. *Barnesiella* and *Feacalibaculum* have been detected in human or mice microbiota [31,32]. *Barnesiella* species have been negatively correlated with the colonization of vancomycin-resistant *Enterococcus faecium* in mice intestines [33] and the relative abundance of the bacterial genera *Faecalibacterium* was significantly decreased in children at risk of asthma [34]. In contrast, dietary xylitol suppressed lipopolysaccharide-induced inflammatory responses in male broiler chickens [35] and has been shown to ameliorate human respiratory syncytial virus infections in mice [36]. Collectively, changes in the fecal microbiota of animals fed xylitol might affect immune responses or colonization of some bacterial species.

Recently, Geidenstam et al. reported that baseline levels of serum xylitol showed an inverse association with a ≥10% weight loss in obese subjects fed low-calorie diet [37]. *Firmicutes phylum* accelerates degradation of food component to supply energy to host, it is, therefore, known as obesity-related bacterial phylum [38]. In our study, the total bacteria/DNA and the relative abundance of *Firmicutes phylum* were increased in the HFD-MX group. Geidenstam and colleague did not examine gut microbiota in their study, human metabolism of xylitol and potential involvement of the gut microbiota could help us to understand the effect of xylitol feeding on human lipid metabolism. 

Xylitol metabolized into xylulose-5-phosphate (X-5-P) is synthesized via the pentose phosphate pathway [39] and activates ChREBP through protein phosphatase 2A [40]; this results in its binding to a specific DNA sequence which induces lipogenesis-related genes which increase lipogenesis from carbohydrates [41]. Daily dietary xylitol at exposure levels ranging between 1.5–4.0 g/kg body weight in combination with a HFD showed a trend towards increased expression of hepatic ChREBP mRNA and a reduction in hepatic triglycerides and total cholesterol as reported in a previous study [8]. These findings suggest xylitol has other functions unrelated to the ChREBP pathway. In the present study, we found that an HFD supplemented with 0.2 g/kg body weight/d of dietary xylitol also induced the increased expression of hepatic ChREBP mRNA and but had a tendency to increase hepatic triglycerides and total cholesterol. The differences between the studies may arise from differences in model species, xylitol dose, and diet which used to characterize the effect of xylitol on lipid metabolism. Because plasma triglyceride level was not increased by HFD feeding in this study, another study that uses another diet (e.g., high-fat high-sucrose diet) which strongly induces hypertriglyceridemia will help to understand the effect of xylitol to alter plasma TG levels. Hepatic total cholesterol in HFD-MX-FMT mice was slightly but significantly higher than that of HFD-FMT mice. Taken together, these changes in the gut microbiota induced by dietary xylitol may potentiate the accumulation of cholesterol and upregulation of hepatic ChREBP. 

In conclusion, we found that 40 and 194 mg/kg body weight/day of dietary xylitol in mice induced gradual changes in gut microbiota, but did not ameliorate HFD-induced dyslipidemia. 

## Figures and Tables

**Table 1:** Oligonucleotide primers.

Primer Name	Sequence (5′–3′)	Reference
Eub338F	ACTCCTACGGGAGGCAGCAG	[20]
Eub518R	ATTACCGCGGCTGCTGG	
HDA1-GC-F	CGCCCGGGGCGCGCCCCGGGCGGGGCGGGGGCACGGGGGGACTCCTACGGGAGGCAGCAGT	[21]
HDA2-R	GTATTACCGCGGCTGCTGGCAC	
Bact934F	GGARCATGTGGTTTAATTCGATGAT	[22]
Bact1060R	AGCTGACGACAACCATGCAG	
Firm934F	GGAGYATGTGGTTTAATTCGAAGCA	
Firm1060R	AGCTGACGACAACCATGCAC	
Prevotella-F	CATGACGTTACCCGCAGAAGAAG	[23]
Prevotella-R	TCCTGCACGCTACTTGGCTG	
mChREBP-F	TCAGCACTTCCACAAGCATC	NM_021455.4
mChREBP-R	GCATTAGCAACAGTGCAGGA	
18sF	AAACGGCTACCACATCCAAG	NR_003278.3
18sR	GGCCTCGAAAGAGTCCTGTA	
mPklr-F	TTGTGCTGACAAAGACTGGC	NM_013631
mPklr-R	CCACGAAGCTTTCCACTTTC	
mFasn-F	TGCCTTCGGTTCAGTCTCTT	NM_007988.3
mFasn-R	GGGCAACTTAAAGGTGGACA	
mScd1-F	CGAGGGTTGGTTGTTGATCT	NM_009127.4
mScd1-R	GCCCATGTCTCTGGTGTTTT	
m II-6-F	CTGATGCTGGTGACAACCAC	NM_031168.2
m II-6-R	TCCACGATTTCCCAGAGAAC	
mTnf-F	AGCCTGTAGCCCACGTCGTA	NM_013693.3
mTnf-R	TCTTTGAGATCCATGCCGTTG	

Eub: Eubacteria (total bacteria), Bact: *Bacteroides*, Firm: *Firmicutes*, ChREBP: Carbohydrate response element binding protein, Pklr: pyruvate kinase liver and red blood cell, Fasn: fatty acid synthase, Scd1: stearoyl-Coenzyme A desaturase 1, Tnf: tumor necrosis factor, II-6: interleukin 6.

**Table 2:** Body weight, organ weight, and plasma parameters of mice fed the control diet or the high-fat diet with or without xylitol.

	Diet				
	CD (*n* = 5)	CD-LX (*n* = 5)	CD-MX (*n* = 5)	HFD (*n* = 5)	HFD-MX (*n* = 6)
Final body weight, g	33.4 ± 0.3	31.2 ± 0.5	32.5 ± 0.8	38.5 ± 1.3	40.5 ± 1.3
Visceral fat, g/kg body weight	15.2 ± 1.2	15.8 ± 1.4	19.4 ± 3.8	43.9 ± 4.5	49.1 ± 2.8
Cecum weight, g/kg body weight	17.7 ± 0.9	16.8 ± 1.3	16.3 ± 0.9	8.9 ± 0.8	8.8 ± 1.4
Hepatic parameters					
Liver, g/kg body weight	48.6 ± 0.7	45.1 ± 1.3	43.0 ± 2.3	43.5 ± 3.8	47.6 ± 3.4
Total cholesterol, mmol/liver	7.5 ± 0.8	7.2 ± 0.4	7.1 ± 0.5	21.3 ± 2.8	33.7 ± 7.3
Triglycerides, mmol/liver	8.8 ± 0.8	11.4 ± 1.3	12.0 ± 1.6	56.0 ± 12.3	74.2 ± 9.2
Plasma parameters					
Total cholesterol, mmol/L	2.0 ± 0.1	2.3 ± 0.1	2.0 ± 0.2	3.6 ± 0.7	4.2 ± 0.3
Triglycerides, mmol/L	1.2 ± 0.1	1.5 ± 0.1	1.4 ± 0.3	1.0 ± 0.1	0.8 ± 0.1

Data represent the mean ± SEM (*n* = 5–6).

**Table 3:** Body weight, organ weight, and plasma parameters of mice fed the high-fat diet following fecal transplantation from mice fed a high-fat diet with or without xylitol.

	Diet	
	HFD-FMT (*n* = 4)	HFD-MX-FMT (*n* = 3)
Final body weight, g	39.5 ± 2.9	40.8 ± 1.4
Visceral fat, g/kg body weight	55.1 ± 6.3	60.6 ± 8.8
Cecum weight, g/kg body weight	6.6 ± 0.8	8.5 ± 1.2
Hepatic parameters		
Liver, g/kg body weight	44.1 ± 1.8	45.1 ± 6.5
Total cholesterol, mmol/liver	82.3 ± 11.1	101.4 ± 16.7
Triglycerides, mmol/liver	23.8 ± 4.6	31.5 ± 3.6
Plasma parameters		
Total cholesterol, mmol/L	4.9 ± 0.1	5.6 ± 0.3 *
Triglycerides, mmol/L	1.1 ± 0.2	1.0 ± 0.2

Data represent the mean ± SEM (*n* = 5). * Significant differences were observed compared with HFD-FMT (*p* < 0.05).
